# Supplementary material for: Amino acid auxotrophies in human gut bacteria are linked to higher microbiome diversity and long-term stability
Source: ISME J. 2023 Oct 27;17(12):2370–80. doi: 10.1038/s41396-023-01537-3 (PMC10689445; doi:10.1038/s41396-023-01537-3)
Supplement: Supplementary file 1 — Supplementary information [file 41396_2023_1537_MOESM1_ESM.pdf]

Supplementary information

Amino acid auxotrophies in human gut bacteria are linked to higher microbiome diversity and long-term stability

Svenja Starke[1], Danielle MM Harris[1,2], Johannes Zimmermann[3,4], Sven Schuchardt[5], Mhmd Oumari[2], Derk Frank[6,7], Corinna Bang[2], Philip Rosenstiel[2], Stefan Schreiber[2,8], Norbert Frey[6,7,#], Andre Franke[2], Konrad Aden[2,8,*], Silvio Waschina[1,*]

[1] Institute of Human Nutrition and Food Science, Nutriinformatics, Kiel University, Kiel, Germany
[2] Institute of Clinical Molecular Biology, Kiel University, Kiel, Germany
[3] Research Group Evolutionary Ecology and Genetics, Zoological Institute, University of Kiel, Kiel, Germany
[4] Max Planck Institute for Evolutionary Biology, Plön, Germany
[5] Fraunhofer Institute for Toxicology and Experimental Medicine (ITEM), Hanover, Germany
[6] Department of Internal Medicine III, University Medical Center Schleswig-Holstein, Kiel, Germany
[7] German Centre for Cardiovascular Research (DZHK), Partner site Hamburg, Kiel, Lübeck, Germany
[8] Department of Internal Medicine I, University Medical Center Schleswig-Holstein, Kiel, Germany
[#] Current Affiliation: Department of Internal Medicine III, University Hospital Heidelberg, Heidelberg, Germany

*Correspondence: s.waschina@nutrinf.uni-kiel.de ; k.aden@ikmb.uni-kiel.de

Supplementary Material and Methods

Reconstruction of genome-scale metabolic models

Genome-scale metabolic models of prokaryotic genomes were reconstructed using *gapseq*(1). In brief, the *gapseq* reconstruction workflow consisted of five steps: (i) Reaction and pathway prediction, (ii) prediction of metabolite cross-membrane transporters, (iii) reconstruction of a draft metabolic network based on the results from *i* and *ii*, (iv) estimation of an organism-specific growth medium-based on the predicted metabolic capabilities, and (v) gap filling of the metabolic network to enable biomass production using flux balance analysis. Model reconstructions were limited to bacterial genomes marked as representative species in the HRGM collection. Further, genomes with an estimated contamination percentage of ≤2% or a completion ≥85% were included. Based on these filters, 3 687 bacterial genomes were subject to metabolic model reconstruction. Among those genomes, 22% are from bacterial isolates, and 78% are metagenome-assembled genomes.

A recent computational study has shown in a systematic analysis of isolate- and metagenome-assembled genomes that the gap-filling medium strongly impacts the auxotrophies predicted by genome-scale metabolic modelling(2). We note that with the reconstruction procedure used in this study, we do not rely on an arbitrarily defined gap-filling medium, which is used for every metabolic network model. Instead, for each draft network, a genome-specific gap-filling medium is predicted (see section "Prediction of a genome-specific gap-filling medium" below for details). In brief, if the medium prediction algorithm of *gapseq* finds a known biosynthetic pathway for a specific amino acid in the draft metabolic network, the amino acid will not be part of the resulting predicted medium,

as the compound is likely not required from the growth environment, thus, also not necessary for subsequent gap filling. In contrast, if the medium prediction algorithm does not detect at least one complete known biosynthetic pathway for a specific amino acid, the respective compound is added to the outcome gap-filling medium based on the rationale that this amino is a putative essential compound that needs to be obtained from the growth environment. However, it is important to note that this case does not directly imply that the organism is auxotrophic for the specific amino acid, as the subsequent gap-filling algorithm might add reactions to the model that complete a biosynthesis route from other compounds in the gap-filling medium to the amino acid. Such reactions are only added in cases where a gene was found in the query genome that displays sequence similarity to a reference gene sequence with the respective enzymatic function but where the sequence similarity was not high enough to pass the threshold of the bitscore 200 to be directly included in the draft network. For details on the gap-filling algorithm implemented in gapseq, please refer to the original gapseq publication(1).

Taken together, the model reconstruction and auxotrophy prediction that we used for the present study do not depend on one gap-filling medium composition that is defined for all organisms but adjusts the medium for each organism based on its genome information and by using the multi-step gap-filling algorithm that is implemented in *gapseq*.

Prediction of a genome-specific gap-filling medium

The genome-scale metabolic network reconstruction process of *gapseq* requires a gap-filling medium for the final gap-filling step (see above). Here, we used a medium prediction feature (module “*gapseq medium*”) of the gapseq software, which can be plugged into the reconstruction workflow between the homology-based generation of a draft metabolic

network and the gap-filling algorithm. We provided the additional command line option “-c cpd00007:0” to ensure that the predicted medium does not contain oxygen (compound identifier: cpd00007). The algorithm for medium prediction tests which pathways and reactions are absent or present in the draft metabolic model. Whether a particular compound is added to the medium is decided using logical expressions that include variables for the presence (TRUE) and absence (FALSE) of pathways and reactions within the design network (see Supplementary Table S6 for all compounds and their logical expressions). For example, the disaccharide lactose (ModelSEED ID: cpd00208), has the logical expression ("LACTOSECAT-PWY" | "LACTOSEUTIL-PWY" | "BGALACT-PWY"), which means, that lactose is added to the medium if one of three known lactose degradation pathways as defined in MetaCyc(3) is already present in the draft network. In particular, for amino acids, the medium prediction module uses a similar approach to the auxotrophy prediction tool GapMind(4), which tests if a known biosynthetic pathway for a specific amino acids exists based on sequence homology. The amino acid biosynthetic pathways that are considered are also those that are defined in MetaCyc(3). For instance, if none of the five known biosynthesis pathways in prokaryotes for lysine (https://metacyc.org/META/NEW-IMAGE?type=PATHWAY&object=LYSINE-SYN) is found, lysine is added to the gap-filling medium.

The medium prediction module of gapseq considered 74 compounds (Supplementary Table S6), including inorganic compounds, carbohydrates, amino acids, other carboxylic acids, and vitamins. Most of those potential nutrients are also compounds that can be found in the growth environment of colonic microorganisms, such as fibers (e.g., pectin, inulin), other dietary compounds (e.g., sulfoquinovose, daidzein), constituents of the mucins (e.g., *N*-acetylneuraminate, *N*-acetylneuraminate) and inorganics (e.g., H_2, H_2S, H_2O).

Besides the enumeration of available nutrients, a gap-filling medium requires their individual maximum uptake rates by the microorganism. The medium prediction implemented in *gapseq* uses rates commonly used in manually curated genome-scale metabolic network models, e.g., 0.1 mmol*gDW^{-1}*hr^{-1} for amino acids or 5 mmol*gDW^{-1}*hr^{-1} for monosaccharides. In the case of oligo- and polysaccharides, the maximum uptake rates are scaled to allow the same uptake rate per subunit (e.g., 5 mmol*gDW^{-1}*hr^{-1} for the monosaccharide D-glucose and 2.5 mmol*gDW^{-1}*hr^{-1} for the disaccharide maltose).

Validation of auxotrophy predictions

To validate our *in silico* predicted auxotrophies, we collected the genome sequences from NCBI RefSeq for 36 bacterial strains for which experimental data were available on amino acid auxotrophies/prototrophies. The majority of these strains were already summarized previously(5). In that study, the authors even list more than the 36 strains we analyzed here. This is because we excluded cases where we could not find a genome assembly of the exact strain used in the referenced experimental study. Moreover, we excluded the entries for species belonging to the genus *Bifidobacterium* since their auxotrophy for cysteine is ambiguous: Some studies report cysteine as an essential nutrient for growth(6,7), while genomic analysis and genome-scale metabolic modeling indicated the presence of cysteine biosynthetic pathways(8,9). This ambiguity most likely stems from the fact that cysteine biosynthesis in *Bifidobacterium* species depends on the available sulfur source(8). Genomic analysis of *Bifidobacterium bifidum* PRL2010, for instance, suggested the strain's inability to use sulfate as a sulfur source, while hydrogen sulfide or methionine could potentially serve as a sulfur source for the biosynthesis of cysteine(6,8).

Genome-scale metabolic models for all 36 strains were reconstructed as described above. Auxotrophies were predicted with the method described in the main manuscript.

In addition to the gapseq-reconstructed models, we also predicted auxotrophies for 20 of the 36 bacterial strains using genome-scale models from the AGORA2 collection(10) (Supplementary Table S2). Auxotrophies were predicted in the same manner as for gapseq models. In contrast to the gapseq models, which can contain only free amino acids and not peptides in the predicted medium, some AGORA2 models have exchange reactions with lower bounds < 0 for dipeptides. In those cases, and for predicting the auxotrophy status for amino acid x, we changed the lower bound to 0 for all exchange reactions of dipeptides involving amino acid x. At the same time, we introduced a new inflow reaction of the non-x amino acid moiety to the model to predict only the essentiality of x and not of the other amino acids in the respective peptides. The sensitivity, specificity, and accuracy of auxotrophy predictions were calculated for *gapseq* models (n=36) and AGORA2 models (n=20).

As an additional auxotrophy prediction validation step, 124 genome-scale metabolic models were reconstructed for bacterial strains that were reported by Price, 2023, to be able to grow in a defined growth medium containing no amino acids(11). Thus, these 124 organisms are known amino acid prototrophs and can be used to estimate the rate of false auxotrophy predictions. The original publication by Price reported 127 genomes of prototrophs; however, 3 of the corresponding genome assemblies (GCF_000014265.1, GCF_000020545.1, GCF_900188395.1) were suppressed on RefSeq at the time we performed the analysis in March 2023. Auxotrophies for the 124 genome assemblies of this prototroph collection were predicted as described above, and results are summarized in Supplementary Table S3.

Metagenome data processing

Metagenomic reads were subject to quality control and filtering using the 'qc' workflow from the metagenome-atlas pipeline tool v2.9.0(12). In detail, reads were (i) deduplicated, (ii) quality filtered, and (iii) decontaminated. Modules from the BBmap suite v37.99 (BBMap - Bushnell B. - sourceforge.net/projects/bbmap/) were used for all three steps. In the deduplication step (i), the BBmap module *clumpify.sh* was used with the parameters "dedupe=t dupesubs=2", which removed duplicate reads with a maximum of 2 substitutions between duplicates. The quality filter (ii) employed the BBmap module *bbduk.sh* with the parameters "hdist=1 ktrim=r mink=8 trimq=10 qtrim=rl minlength=51 maxns=-1 minbasefrequency=0.05" and otherwise default options. This quality filter trimmed reads from the right if adapter sequences were detected, trimmed reads on both sides from the first base with a quality score below 10, removed sequences that were shorter than 51 bp after trimming, removed sequences with ambiguous base calls (i.e., "N"s), and removed reads if any base had a frequency of less than 5%. Finally, reads that are likely contaminations from the human host genome or Illumina PhiX sequences were removed using the BBmap module *bbsplit.sh* using the option "maxratio=0.65" and otherwise default parametrization. This tool tested if specific reads mapped to the host genome or PhiX sequences based on sequence similarity and mapped reads were discarded from the sample's fastq files. For the decontamination step (iii), the human reference genome assembly 'Genome Reference Consortium Human Build 38' (GRCh38) was used, in which low entropy regions (entropy < 0.7) were masked using the *bbmask.sh* tool within the BBmap suite. Moreover, regions that display high similarity to prokaryotic rRNA genes were additionally masked. To this end, prokaryotic small and large subunit rRNA gene sequences

were retrieved from SILVA version 138.1(13) and shredded into shorter (80 bp) sequences with 40 bp overlaps using *shred.sh*. Shredded sequences were aligned to GRCh38 with a minimum identity of 85% and maximum indel length of 2 bp. Regions in GRCh38 with alignment hits were masked.

As mentioned in the main manuscript, we used the Human Reference Gut Microbiome ‘HRGM’ catalog(14) as reference genomes for quantifying representative microbial genomes in the metagenomic data sets. We had chosen this collection, as it was the latest published human gut microorganism genome collection when we were finalizing the results of the present study. Furthermore, the HRGM collection contains 780 species-level representative genomes, which were absent in previous genome collections and assembled from metagenome samples from before under-represented Asian countries, namely Korea, Japan, and India. For each metagenome sample, the relative abundance of HRGM genomes was estimated using coverM(15) v0.6.1 with default parametrization of the module `coverm genome`.

Targeted metabolomics of blood samples

Serum samples were collected using serum s-monovette (9ml, Sarstedt, Germany). Samples were incubated upright at RT for 30 min. and centrifuged (10 min., 2000 x g). Serum was aliquoted in 500µl tubes and stored at -80°C. Metabolite quantification for serum was performed by liquid chromatography tandem mass spectrometry (LC-MS-MS) using the MxP Quant 500 kit (Biocrates Life Sciences AG, Innsbruck, Austria) according to the manufacturer's instructions. The MxP Quant 500 kit simultaneously measures 630 metabolites covering 14 small molecule and 12 different lipid classes. It combines flow injection analysis tandem mass spectrometry (FIA-MS/MS) using SCIEX 5500 QTrap mass

spectrometer (SCIEX, Darmstadt, Germany) for lipids and liquid chromatography tandem mass spectrometry (LC-MS/MS) using Agilent 1290 Infinity II liquid chromatography (Santa Clara, CA, USA) coupled with a SCIEX 5500 QTrap mass spectrometer for small molecules using multiple reaction monitoring (MRM) to detect the analytes. Data evaluation for serum metabolite concentrations and quality assessment was performed with the software SCIEX Analyst software (Version 1.7.2) and the MetIDQ™ software package (Oxygen-DB110-3023), which is an integral part of the MxP Quant 500 kit.

For downstream statistical analysis, the serum metabolome data were pre-processed by imputing missing specific values using a random forests approach as implemented in the R-package 'missForest' and the function with the same name in default parametrization (16). This imputation was limited to missing values for metabolites, which have less than 20% missing values across the data set.

With a partial Spearman correlation, the association between the frequency of auxotrophic bacteria and serum metabolites and other hematology parameters from the DZHK cohort was evaluated (17). We adjusted for the potential confounders sex, age, and BMI. *p* values were corrected for multiple testing using the False Discovery Rate (FDR) method.

Supplementary Figures

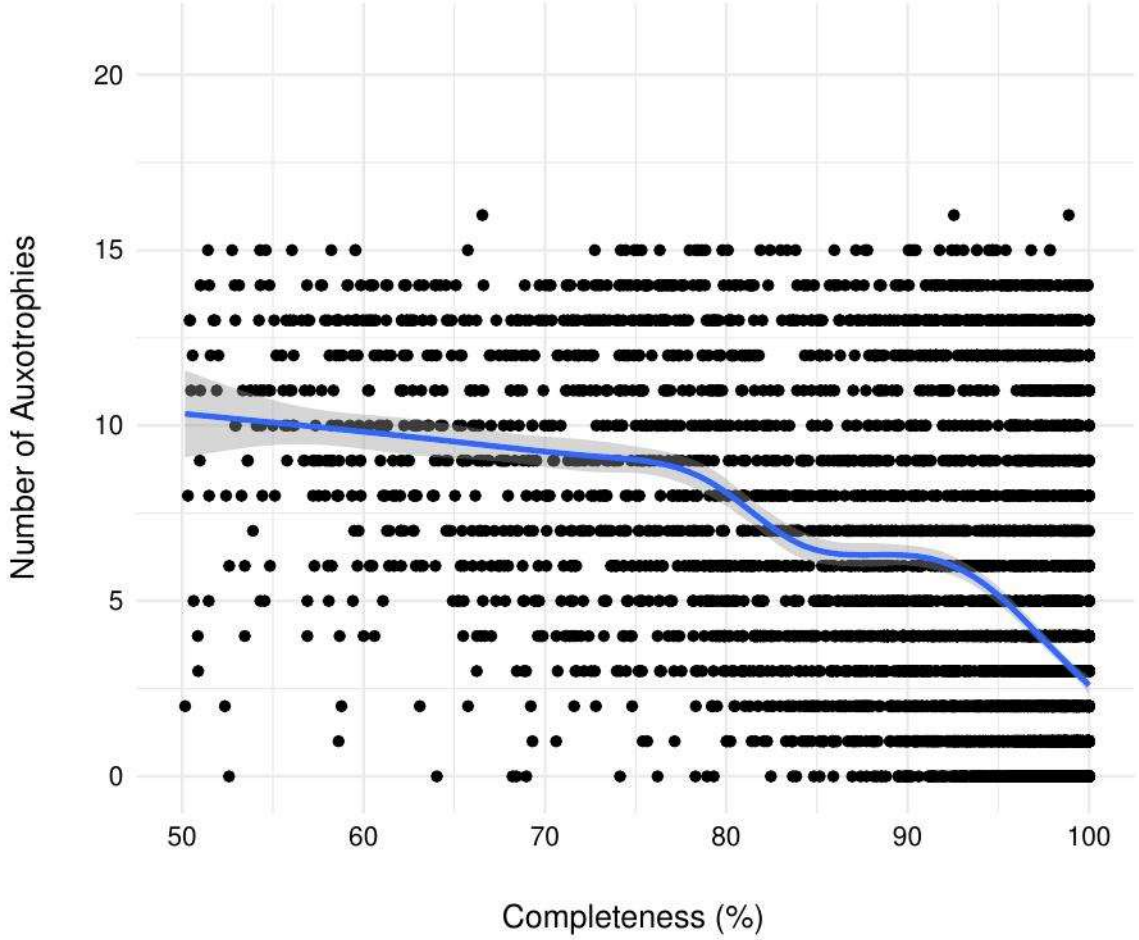

Supp. Figure S1: Estimated genome completeness and the predicted number of auxotrophies for 3 687 genomes of representative species from the Human Reference Gut Microbiome (HRGM) collection*(14)*. The blue line shows the regression line ([illegible]= -0.50, p ≤ 2.2e-16).

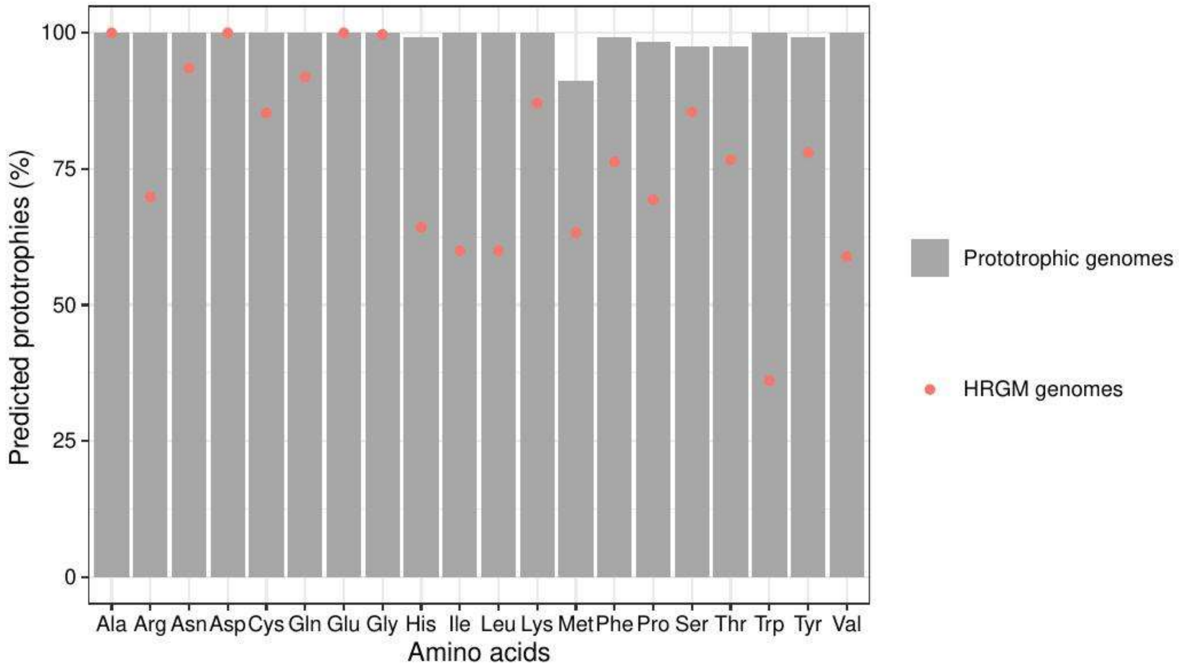

Supp. Figure S2: Percentage of *in silico* predicted prototrophies with metabolic modeling in 124 genomes known to be prototrophic(11) from laboratory experiments (grey bars). The red dots indicate the frequency of prototrophies among 3 687 genomes from human gut bacteria(14).

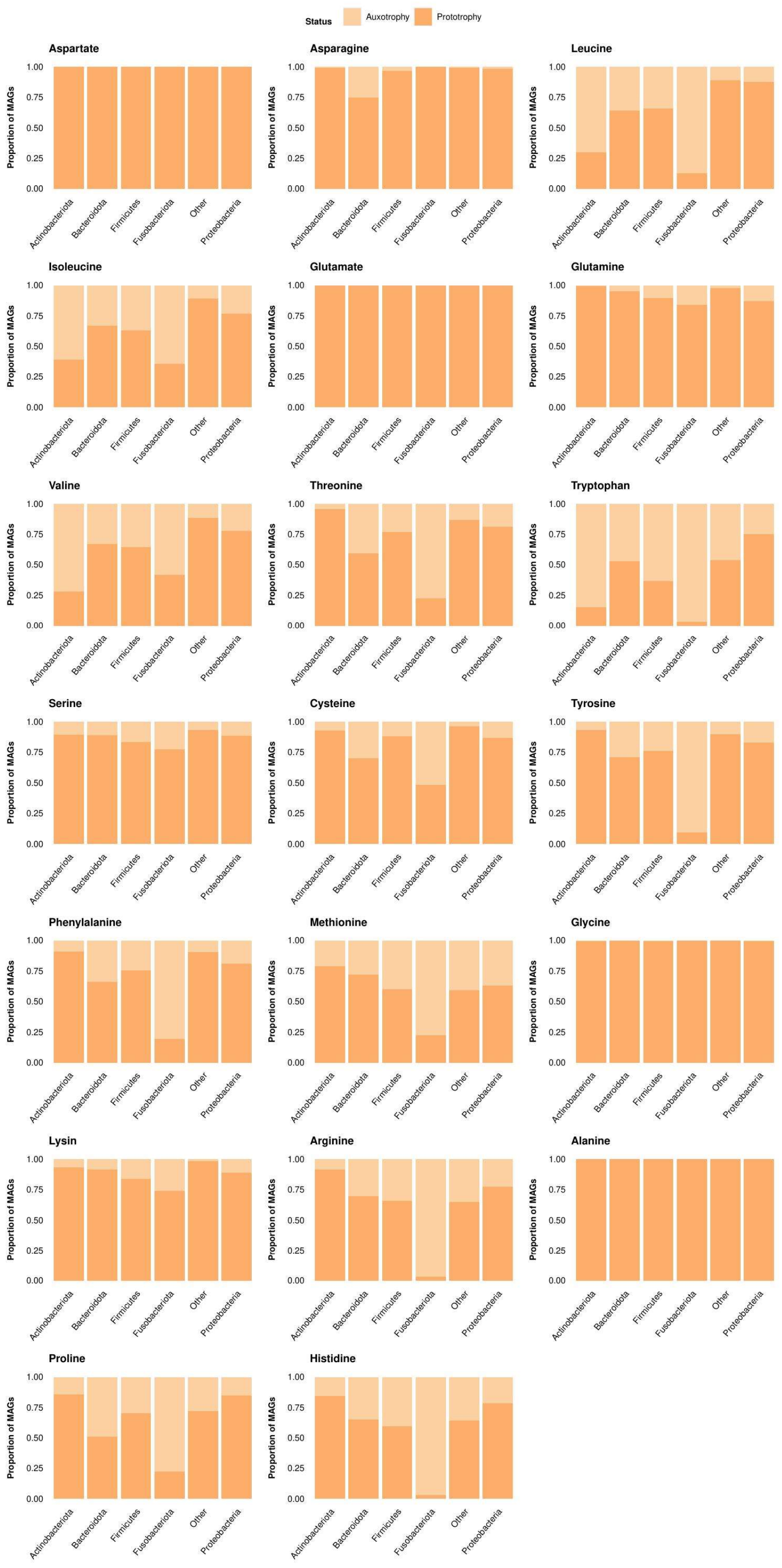

Supp. Figure S3: Overview of the proportions of auxotrophy to prototrophy genomes per phylum from the HRGM catalog(14).

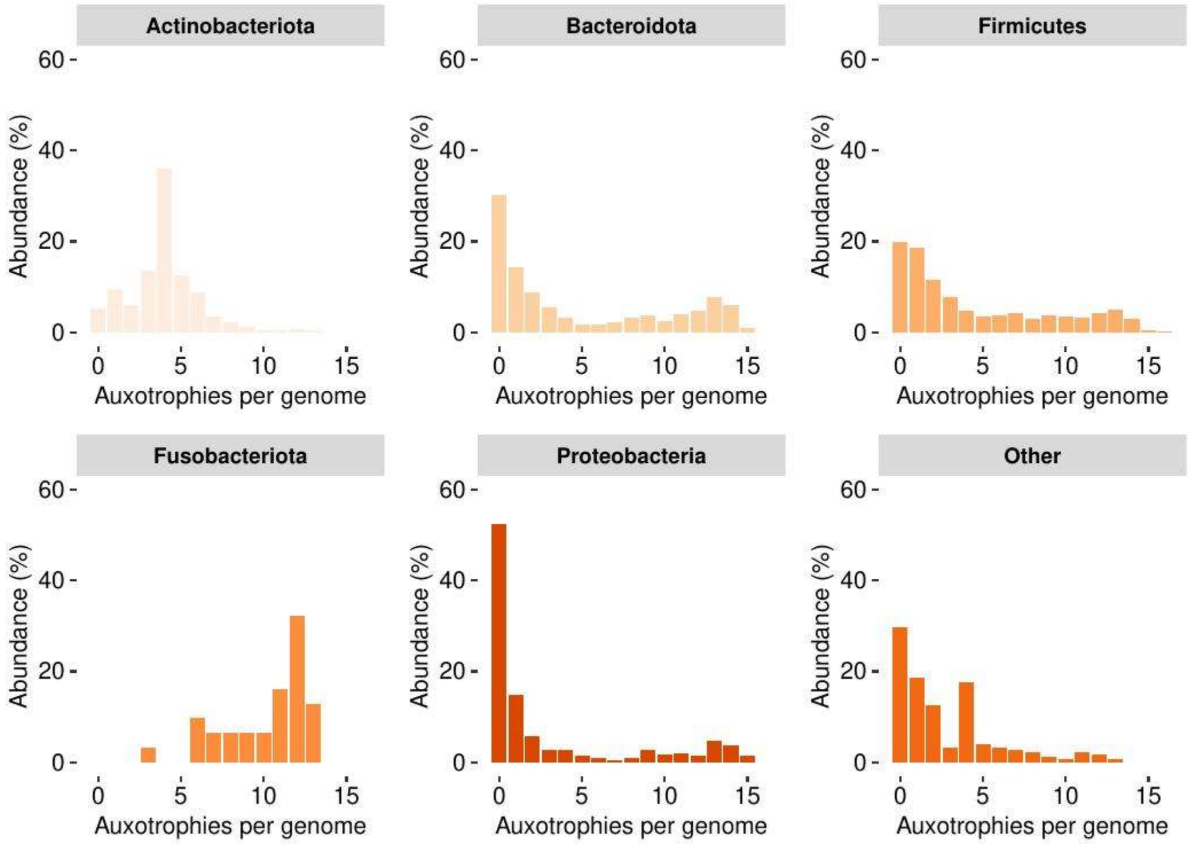

Supp. Figure S4: Number of auxotrophies for every phylum. Other is a category that combines different phyla with a lower abundance in the overall HRGM catalogue(14) and for a reduction of complexity.

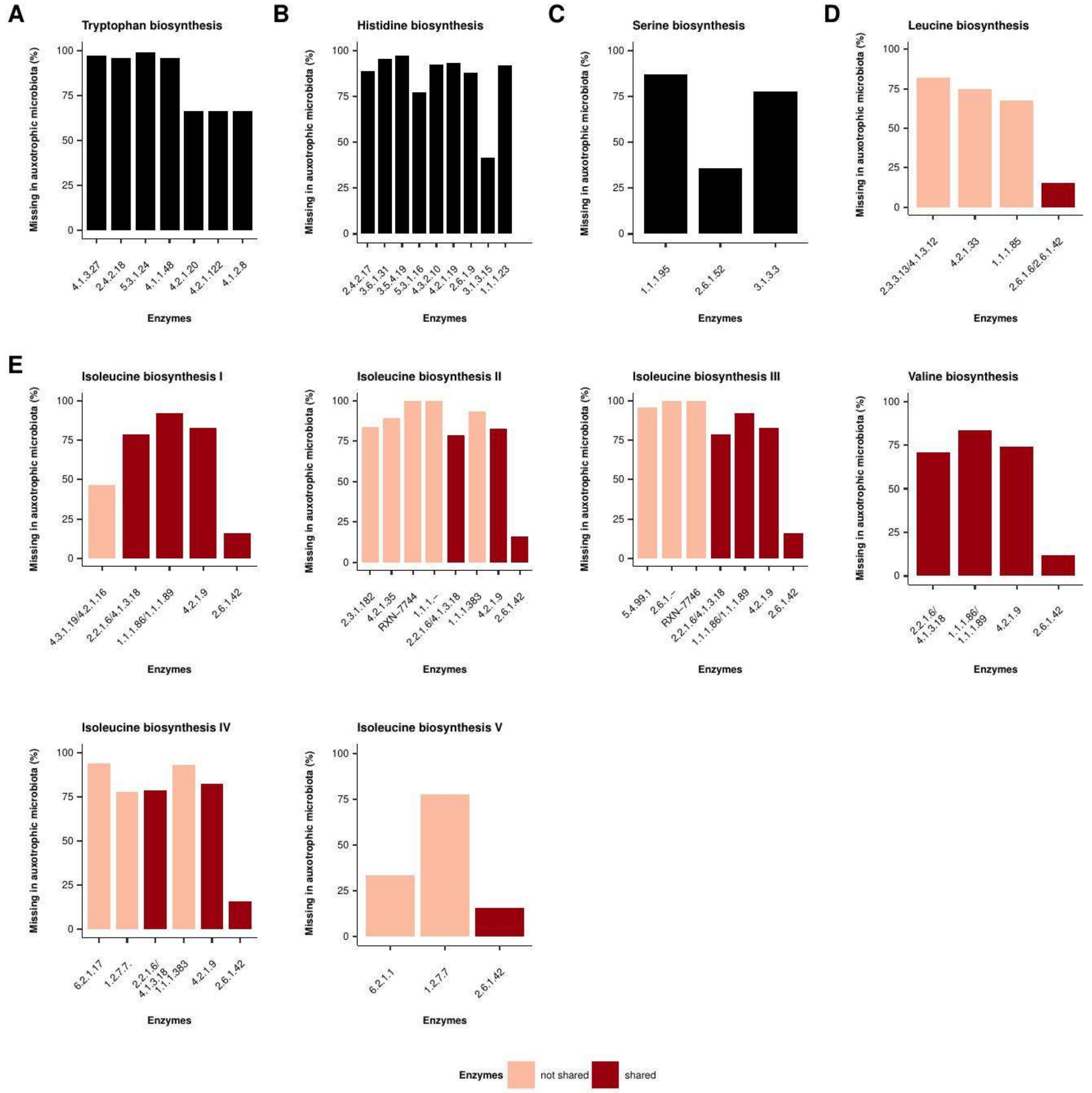

Supp. Figure S5: The bar plots display the abundance of missing enzymes in the biosynthesis pathways for several amino acids and BCAA biosynthesis pathways in auxotrophic bacteria. The order of the enzymes in the bar plots represents the one in the pathway. (A) Missing enzymes in the tryptophan pathway of tryptophan auxotrophic bacteria, (B) Missing enzymes in the histidine pathway of histidine auxotrophic bacteria, (C) Missing enzymes in the chorismate pathway of chorismate auxotrophic bacteria, (D) Missing enzymes in the serine pathway of serine auxotrophic bacteria, (E) Comparison of the BCAA pathways of

isoleucine, leucine, and valine auxotrophic bacteria, the colors indicate which enzymes are shared in the biosynthesis pathways, the definition of the pathways are based on MetaCyc.

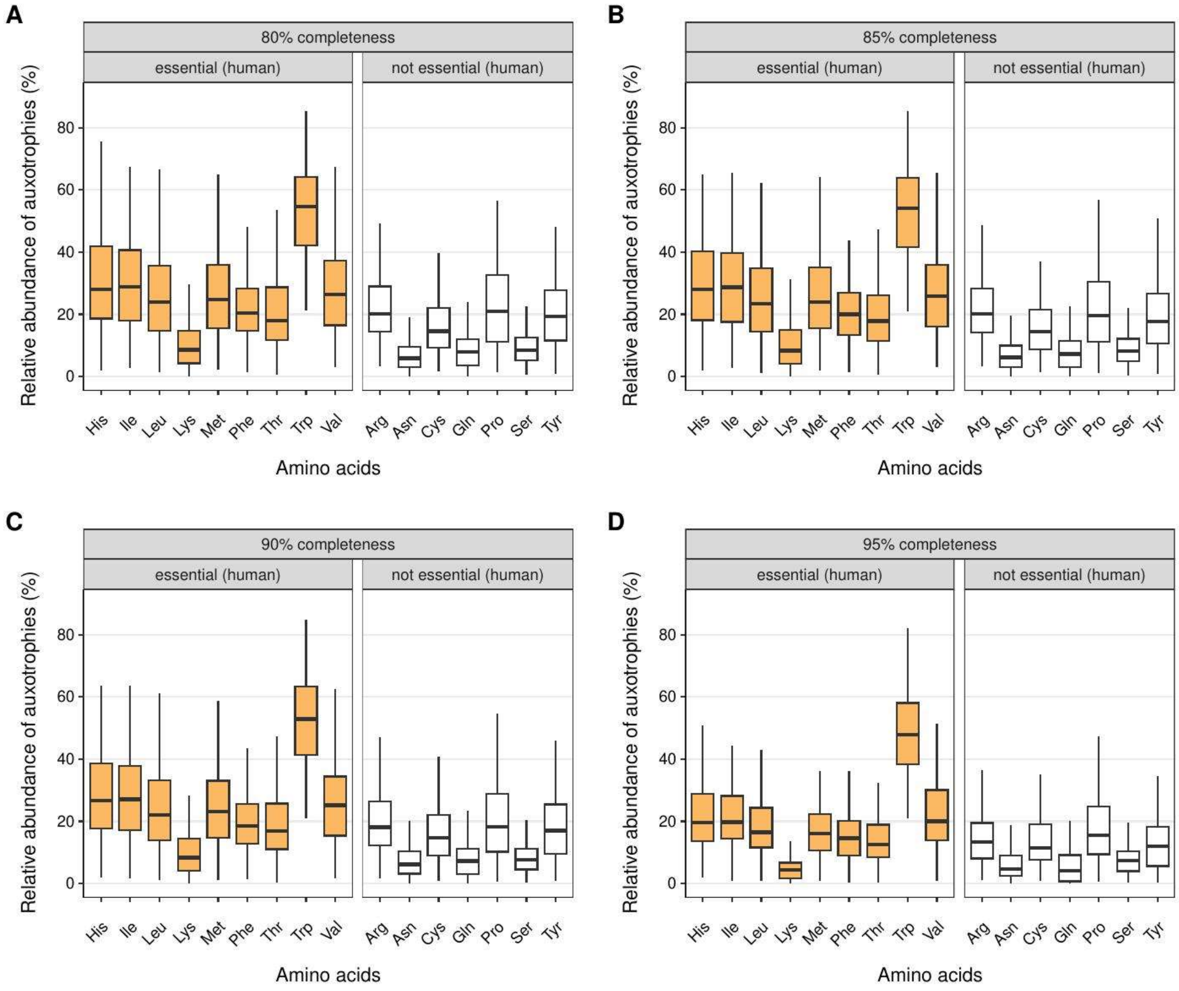

Supp. Figure S6: Relative abundance of predicted amino acids auxotrophs depending on the completeness cutoff for reference genomes from the HRGM catalog. Four different genome completeness cutoffs were tested: 80% (A), 85% (B, same as Figure 4A), 90% (C), and 95% (D). The results indicate that the distribution of the relative abundance of predicted amino acid auxotrophies was stable with respect to the chosen completeness cut-off for reference genome filtering.

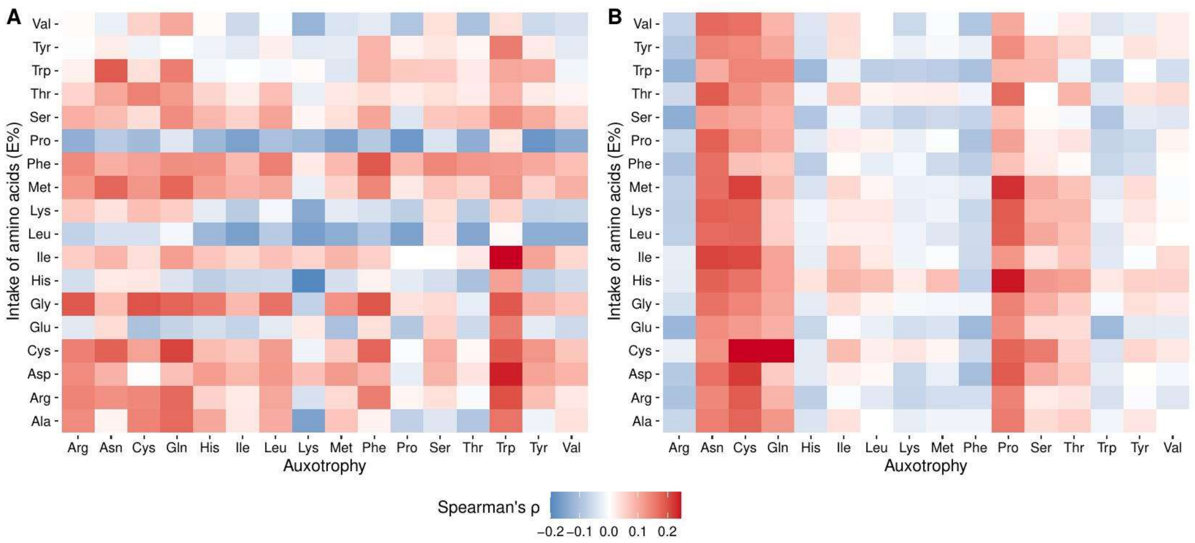

Supp. Figure S7: Spearman correlation between the dietary intake of amino acids and the frequency of amino acid auxotrophic bacteria in the gut microbiomes, (A) at the beginning of the study, (B) at the end of the study (3 years later). No statistically significant associations were found (FDR-corrected *p* value >0.05).

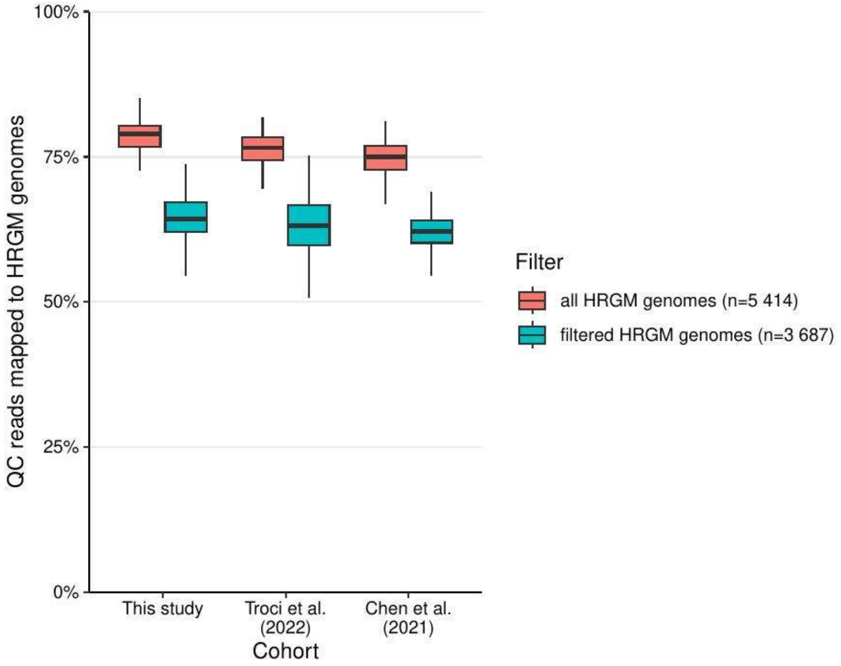

Supp. Figure S8: Percentage of quality-controlled metagenomic reads from three cohorts (this study, Troci *et al*. 2022 (18), and Chen *et al*. 2021 (19)) to reference genomes from the HRGM catalog.

References

1. Zimmermann J, Kaleta C, Waschina S. gapseq: informed prediction of bacterial metabolic pathways and reconstruction of accurate metabolic models. Genome Biol. 2021 Dec;22(1):81.

2. Borer B, Magnúsdóttir S. The media composition as a crucial element in high-throughput metabolic network reconstruction. Interface Focus. 2023 Apr 6;13(2):20220070.

3. Caspi R, Billington R, Fulcher CA, Keseler IM, Kothari A, Krummenacker M, et al. The MetaCyc database of metabolic pathways and enzymes. Nucleic Acids Res. 2018 Jan 4;46(D1):D633–9.

4. Price MN, Deutschbauer AM, Arkin AP. GapMind: Automated Annotation of Amino Acid Biosynthesis. Hallam SJ, editor. mSystems. 2020 Jun 30;5(3):e00291-20.

5. Ashniev GA, Petrov SN, Iablokov SN, Rodionov DA. Genomics-Based Reconstruction and Predictive Profiling of Amino Acid Biosynthesis in the Human Gut Microbiome. Microorganisms. 2022 Apr;10(4):740.

6. Ferrario C, Duranti S, Milani C, Mancabelli L, Lugli GA, Turroni F, et al. Exploring Amino Acid Auxotrophy in Bifidobacterium bifidum PRL2010. Front Microbiology. 2015 Nov;6:1331.

7. Veda M, Nakamoto S, Nakai R, Takagi A. Establishment of a defined minimal medium and isolation of auxotrophic mutants for *Bifidobacterium bifidum* Es 5. J Gen Appl Microbiol. 1983;29(2):103–14.

8. Schöpping M, Gaspar P, Neves AR, Franzén CJ, Zeidan AA. Identifying the essential nutritional requirements of the probiotic bacteria Bifidobacterium animalis and Bifidobacterium longum through genome-scale modeling. npj Syst Biol Appl. 2021 Dec 9;7(1):47.

9. Lee JH, O'Sullivan DJ. Genomic Insights into Bifidobacteria. Microbiol Mol Biol Rev. 2010 Sep;74(3):378–416.

10. Heinken A, Hertel J, Acharya G, Ravcheev DA, Nyga M, Okpala OE, et al. Genome-scale metabolic reconstruction of 7,302 human microorganisms for personalized medicine. Nat Biotechnol. 2023 Sept;41:1320-1331.

11. Price M. Erroneous predictions of auxotrophies by CarveMe. Nat Ecol Evol. 2023 Feb;7(2):194–5.

12. Kieser S, Brown J, Zdobnov EM, Trajkovski M, McCue LA. ATLAS: a Snakemake workflow for assembly, annotation, and genomic binning of metagenome sequence data. BMC Bioinformatics. 2020 Dec;21(1):257.

13. Quast C, Pruesse E, Yilmaz P, Gerken J, Schweer T, Yarza P, et al. The SILVA ribosomal RNA gene database project: improved data processing and web-based tools. Nucleic Acids Res. 2012 Nov 27;41(D1):D590–6.

14. Kim CY, Lee M, Yang S, Kim K, Yong D, Kim HR, et al. Human reference gut microbiome catalog including newly assembled genomes from under-represented Asian metagenomes. Genome Med. 2021 Dec;13(1):134.

15. Woodcroft BJ. CoverM [Internet]. 2023 [cited 2023 Aug 20]. Available from: https://github.com/wwood/CoverM

16. Daniel J. Stekhoven. missForest: Nonparametric Missing Value Imputation using Random Forest. Bioinformatics. 2012;28(1):112–8.

17. Liu Q, Li C, Wanga V, Shepherd BE. Covariate-adjusted Spearman's rank correlation with probability-scale residuals: Covariate-Adjusted Spearman's Rank Correlation. Biometrics. 2018 Jun;74(2):595–605.

18. Troci A, Rausch P, Waschina S, Lieb W, Franke A, Bang C. Long-Term Dietary Effects on Human Gut Microbiota Composition Employing Shotgun Metagenomics Data Analysis. Mol Nutr Food Res. 2022 Jun 27;e2101098.

19. Chen L, Wang D, Garmaeva S, Kurilshikov A, Vich Vila A, Gacesa R, et al. The long-term genetic stability and individual specificity of the human gut microbiome. Cell. 2021 Apr;184(9):2302-2315.e12.
